# Clinical Case Scenarios in the Management of Non-diabetic Chronic Kidney Diseases

**DOI:** 10.7759/cureus.75770

**Published:** 2024-12-15

**Authors:** Sharad Sheth

**Affiliations:** 1 Nephrology, Kokilaben Dhirubhai Ambani Hospital and Medical Research Institute, Mumbai, IND

**Keywords:** dapagliflozin, ndkd, non-diabetic kidney disease, proteinuria, renoprotective, sglt2i, sodium-glucose co-transporter 2 inhibitor

## Abstract

Research conducted in India has shown that there is a high prevalence of non-diabetic kidney disease (NDKD) among Indian patients. Sodium-glucose co-transporter 2 inhibitors (SGLT2i) are emerging as potential treatments for preventing the progression of chronic kidney disease to advanced stages, regardless of their anti-diabetic effects. Dapagliflozin, which has been approved by the Central Drugs Standard Control Organization, is the SGLT2i drug class approved for use in both DKD and NDKD patients. Taking this validation into account, the case series was planned to showcase four different real-life case studies in an Indian clinical setting, which involved the utilization of dapagliflozin in different patient profiles with NDKD. The real-world cases substantiate the renoprotective effects of dapagliflozin as seen by changes in the estimated glomerular filtration rate levels and proteinuria post-treatment. These case scenarios highlight the benefits of reducing renal risk factors in patients with NDKD. Notably, in all the cases, the risk of renal disease progression was delayed in NDKD patients with dapagliflozin.

## Introduction

The burden of chronic kidney disease (CKD) is rapidly increasing both globally and in India. The Screening and Early Valuation of Kidney Disease study was conducted on approximately 6,000 Indian adult subjects from 21 centers to determine the prevalence of CKD. The CKD prevalence of 17.4% was reported to be 17-fold higher in Indian patients as compared to other Indian community-based studies [1].

CKD is considered a crucial risk factor for cardiovascular diseases (CVDs), which account for high premature mortality rates and disability-adjusted life years [2]. The Global Burden of Disease study estimated an alarming 20% increase in mortality rates globally due to CKD in the year 2019 as compared to the year 2010 [3]. Similarly, a 38% steep rise in mortality rates due to CKD was reported in Indian patients from the years 2001-03 to 2010-13 [4].

The major clinical challenges often faced in India are missed opportunities for secondary and tertiary prevention of CKD, as Indian patients usually seek late medical attention when symptoms arise in the course of the disease. Generally, the diagnosis of kidney disease is possible in earlier stages only during routine health check-ups or when kidney function tests are performed for other health conditions [2].

An observational study was conducted on Indian patients to analyze the spectrum of kidney involvement in 5,485 renal biopsies in the form of diabetic kidney disease (DKD) or non-DKD (NDKD). Histological features of DKD in 31% of patients, pure NDKD in 49% of patients, and NDKD plus DKD lesions in 20% of patients who underwent biopsy were reported. This study concluded an increased prevalence rate of NDKD in Indian patients [5].

Another prospectively performed renal biopsy study conducted on 110 patients highlighted that the most common diagnosis reported in Indian patients with NDKD was immunoglobulin A (IgA) nephropathy. Similarly, in the mixed kidney disease group, the most common subtype of NDKD was reported to be focal segmental glomerulosclerosis (FSGS) in Indians [6].

Angiotensin-converting enzyme inhibitors (ACEIs) and angiotensin receptor blockers (ARBs) are the main pharmacologic therapies to delay CKD progression. These therapies have renal hemodynamic effects to decrease intraglomerular pressure and proteinuria. Currently, sodium-glucose co-transporter 2 inhibitors (SGLT2i) are emerging as potential treatments to prevent CKD progression to later stages independent of their anti-diabetic effects [7]. Based on the Central Drugs Standard Control Organization’s approval, dapagliflozin is approved for use in DKD and NDKD patients [8,9].

Role of SGLT2i in NDKD

The drug class of SGLT2i is proven to reduce the risk of sustained loss of kidney function, decline in estimated glomerular filtration rate (eGFR), worsening of albuminuria, new end-stage kidney disease (ESKD), and renal deaths [10]. Compelling evidence shows that SGLT2i also reduces CKD progression in non-diabetic patients with variable degrees of proteinuria, suggesting that SGLT2 activation is involved in CKD pathogenesis irrespective of its diabetes status [11].

The Dapagliflozin and Prevention of Adverse Outcomes in the Chronic Kidney Disease (DAPA-CKD) study was the first clinical study to demonstrate a risk reduction in all-cause mortality and improvement in kidney disease outcomes with SGLT2i in CKD patients irrespective of their diabetes status. In this study, adult patients with or without type 2 diabetes mellitus (T2DM), eGFR 25-75 mL/min/1.73 m^2^, and albumin-to-creatinine ratio 200-5000 mg/g (20-500 mg/mmol) and who were on a stable tolerated dose of either ACEI or ARB were either randomized to dapagliflozin (10 mg daily) or placebo [8,10].

Growing evidence suggests that the positive effects of SGLT2i are independent of their anti-diabetic effects, which may be mediated by natriuresis and glucose-induced osmotic diuresis, leading to a decrease in intraglomerular pressure. These beneficial renoprotective effects are mediated by a decrease in glomerular pressure due to constriction of pathologically dilated afferent arterioles in both DKD and NDKD patients [8]. With this validation in mind, this case series was planned to share unique real-world case scenarios managed with dapagliflozin in diverse patient profiles with NDKD in an Indian clinical setting.

## Case presentation

Case 1: IgA nephropathy with hypertension and stage 1 CKD

A 53-year-old female patient with a known case of hypertension was diagnosed with IgA nephropathy eight years ago and stage 1 CKD 1.5 years ago. The patient was receiving telmisartan 40 mg/day and was advised to control fluid and protein intake.

On the general examination, the patient weighed 80 kg, had a pulse rate of 68 beats/min, and a blood pressure reading of 150/90 mmHg. No abnormalities were detected during the systemic examination of the patient's cardiovascular, respiratory, and gastrointestinal systems. However, in urine analysis, hematuria was noticed. The laboratory findings are mentioned in Table 1.

**Table 1:** Laboratory findings for case 1 IgA: immunoglobulin A, eGFR: estimated glomerular filtration rate, CKD: chronic kidney disease

IgA nephropathy with hypertension and stage 1 CKD		
eGFR	60 mL/min/1.73 m^2 ^	Normal range: ≥90 mL/min/1.73 m^2^
Protein	4 g/day	Normal range: <150 mg/day
Serum creatinine	1.8 mg/dL	Normal range: 0.7 to 1.3 mg/dL
Complete blood count and lipid profile	Within normal limits	

Given the patient's stage 2 CKD, it was determined that a renoprotective drug would likely be beneficial. Consequently, the patient was prescribed dapagliflozin 10 mg/day in addition to her medication regimen of telmisartan and fluid and protein intake restriction.

After a 12-month follow-up, improvements were observed in the patient's progress. The patient had successfully achieved a weight reduction of 2 kg, and her blood pressure was well-controlled. The eGFR and serum creatinine levels were reported to be within normal limits, indicating improved kidney function. Most notably, the proteinuria had significantly reduced from 4 g/day to 2 g/day. The reduction in proteinuria and improvement in eGFR demonstrate the renoprotective effects of dapagliflozin, affirming its effectiveness in managing IgA nephropathy and stage 2 CKD in this patient. No adverse events were reported by this patient during treatment and follow-up duration.

Overall, this case scenario highlights the successful effect of dapagliflozin as a renoprotective drug in a 53-year-old female patient with IgA nephropathy and stage 2 CKD.

Case 2: IgA nephropathy with hypertensive CKD and dapagliflozin as adjunct therapy

In this case scenario, a 53-year-old female patient was diagnosed with CKD due to IgA nephropathy and hypertension. Over the past four years, the patient has progressed from CKD stage 1 to stage 2. This patient did not have diabetes. Her management plan included losartan 40 mg/day, restricted salt, fluids, and protein intake.

On examination, the patient weighed 58 kg, had a pulse rate of 68 beats/min, and a blood pressure reading of 135/90 mmHg. Her systemic examination was unremarkable. The laboratory workup results are depicted in Table 2.

**Table 2:** Laboratory findings for case 2 IgA: immunoglobulin A, eGFR: estimated glomerular filtration rate, CKD: chronic kidney disease

IgA nephropathy with hypertensive CKD and dapagliflozin as adjunct therapy		
eGFR	70 mL/min/1.73 m^2 ^	Normal range: ≥90 mL/min/1.73 m^2^
Proteins	1 g/day	Normal range: <150 mg/day
Complete blood count and lipid profile	Within normal limits	

Despite being treated with an ARB such as losartan, the patient's CKD continued to progress. Consequently, there was a clear need for an additional drug with proven renoprotective effects. To address this, dapagliflozin 10 mg/day was added to the patient's medication regimen.

After a follow-up period of four months, positive changes were observed in the patient's condition. The patient's weight reduced by 2 kg, and her blood pressure remained under control. The eGFR improved from 70 mL/min/1.73 m^2^ to 75 mL/min/1.73 m^2^, indicating a positive response to the treatment. Additionally, the protein levels in the urine had reduced.

Overall, this case highlights the addition of dapagliflozin as an effective renoprotective drug in the management of a patient with CKD due to IgA nephropathy. The patient showed improvements in kidney function, blood pressure, and a reduction in proteinuria after incorporating dapagliflozin along with ARB losartan. The results further support the use of dapagliflozin as an adjunct therapy for patients with CKD with IgA nephropathy, providing improved outcomes in this patient population.

Case 3: FSGS with hypertension and non-adherence to medication regimen

A 61-year-old female patient was diagnosed with FSGS, and her CKD progressed from grade 2 to grade 3 in the last four years. The patient has a history of hypertension and CVD spanning five years. The patient was prescribed antihypertensives and aspirin but was not compliant with the medication regimen.

At the time of assessment, she weighed 60 kg and had a pulse rate of 76 beats/min and a blood pressure reading of 160/110 mmHg. Her systemic examination was unremarkable. The ECG and lipid panel were within normal limits. The laboratory findings are depicted in Table 3, suggesting stage 3 kidney disease.

**Table 3:** Laboratory findings for case 3 FSGS: focal segmental glomerulosclerosis, eGFR: estimated glomerular filtration rate, UACR: urinary albumin-to-creatinine ratio

FSGS with hypertension and non-adherence to medication regimen		
eGFR	50 mL/min/1.73 m^2 ^	Normal range: ≥90 mL/min/1.73 m^2^
UACR	400 mg/g	Normal range: <30 mg/g

Given the patient's CKD, dapagliflozin 10 mg/day was prescribed in addition to the current medication regimen. The patient received counseling for treatment adherence.

The patient was followed up regularly, and at a six-month follow-up, positive changes were observed in the patient's condition. The patient adhered to dapagliflozin and lifestyle modifications, and her weight was reduced by 5 kg. Additionally, her blood pressure reduced to 140/90 mmHg from 160/110 mmHg. Furthermore, the UACR values decreased to 350 mg/g from 400 mg/g. This improvement in blood pressure and UACR was crucial in reducing the progression of CKD. The patient’s eGFR was 48 mL/min/1.73 m^2^.

This case highlights the importance of patient adherence to medication regimens, particularly in the management of CKD. The addition of dapagliflozin to antihypertensive and aspirin therapy proved beneficial in improving kidney function and reducing albuminuria. These positive outcomes emphasize the potential role of dapagliflozin as an adjunct therapy in the management of FSGS-related CKD, particularly in patients with a history of hypertension and CVD. Continued adherence to the prescribed medications and regular follow-up visits will be key in maintaining and further improving the patient's renal health.

Case 4: dapagliflozin and corticosteroid therapy in FSGS-related CKD

This is a case of a 60-year-old female patient diagnosed with FSGS five years back. Two years ago, she was diagnosed with stage 4 CKD, accompanied by hypertension. To manage her hypertension, she was prescribed telmisartan 40 mg/day two years back.

At the time of assessment, the patient weighed 80 kg, and her vital signs were stable with a pulse rate of 78 beats/min and a blood pressure reading of 150/90 mmHg. On examination, the patient's respiratory and gastrointestinal systems were unremarkable. The laboratory workup is shown in Table 4.

**Table 4:** Laboratory findings for case 4 FSGS: focal segmental glomerulosclerosis, eGFR: estimated glomerular filtration rate, CKD: chronic kidney disease

Dapagliflozin and corticosteroid therapy in FSGS-related CKD		
eGFR	25 mL/min/1.73 m^2 ^	Normal range: ≥90 mL/min/1.73 m^2^
Proteins	>7 g/day	Normal range: <150 mg/day
Complete blood count and lipid profile	Within normal limits	

To address the patient's condition, a treatment plan included dapagliflozin 5 mg/day along with corticosteroid therapy, with the aim of improving renal function and reducing proteinuria. The patient was diligently followed thereafter.

During the follow-up visit after six months, the patient reported a significant weight reduction of 5 kg, reflecting a positive step toward progressing to a normal BMI. Furthermore, her blood pressure was under control, and her eGFR level showed improvement, increasing to 28 mL/min/1.73 m^2^, indicating a positive response to the treatment. Additionally, the patient showed a reduction in proteinuria, decreasing to 5 g/day.

This case highlights the potential benefits of combining dapagliflozin and corticosteroid therapy in managing FSGS-related CKD. The observed weight reduction, improved blood pressure control, and favorable changes in eGFR and proteinuria levels signify a promising outcome.

**Table 5:** Short summary of all cases FSGS: focal segmental glomerulosclerosis, eGFR: estimated glomerular filtration rate, CKD: chronic kidney disease, IgA: immunoglobulin A

	Case 1	Case 2	Case 3	Case 4
Age/gender	53/F	53/F	61/F	60/F
Comorbidities	IgA nephropathy hypertension	IgA nephropathy hypertension	FSGS hypertension and CVD	FSGS hypertension
Stage of CKD	Stage 1	Stage 2	Stage 3	Stage 4
eGFR pre-dapagliflozin therapy	60 ml/min/1.73 m^2^	70 ml/min/1.73 m^2^	50 ml/min/1.73 m^2^	25 ml/min/1.73 m^2^
eGFR post-dapagliflozin therapy	>90 ml/min/1.73 m^2^ (within normal limits)	70 ml/min/1.73 m^2^	48 ml/min/1.73 m^2^	28 ml/min/1.73 m^2^
Proteinuria pre-dapagliflozin treatment	4 gm/day	1 gm/day	UACR - 400 mg/g	>7 gm/day
Proteinuria post-dapagliflozin treatment	2 gm/day	Reduced	UACR - 350 mg/g	5 gm/day

## Discussion

The real-world cases presented here substantiate the renoprotective effects of dapagliflozin as seen by changes in the eGFR levels and proteinuria post-treatment (Table 5). Notably, the risk of renal disease progression was delayed in NDKD patients.

SGLT2i has the potential to be a disease-modifying intervention to prevent a CKD risk progression irrespective of diabetes status in the EMPA-CKD (Empagliflozin in Patients With CKD) study and DAPA-CKD trials. Moreover, both the renoprotective and cardioprotective effects of SGLT2i were extended in CKD from T2DM to NDKD patients in the DAPA-CKD study [8,10].

An initial decrease in eGFR of approximately 4-5 mL/min/1.73 m^2^ often referred to as GFR "dip,” is often reported within the first two to three weeks of administering SGLT2i therapy [8,10]. However, this eGFR dip reported immediately after starting SGLT2i probably highlights its protective mechanism and is not associated with any safety concerns. Compelling data from the EMPA-REG (Empagliflozin Cardiovascular Outcome Event Trial in Type 2 Diabetes Mellitus Patients) outcome study, VERTIS-CV (Cardiovascular Outcomes Following Ertugliflozin Treatment in Type 2 Diabetes Mellitus Participants With Established Cardiovascular Disease), and CREDENCE (Canagliflozin and Renal Outcomes in Type 2 Diabetes and Nephropathy) studies, and consistent safety data in real-world evidence studies confirm that this eGFR dip is unrelated to progressive long-term kidney function loss and should not have any impact on the continuation of SGLT2i therapy [12].

The DAPA-CKD study (4,304 CKD patients with or without T2DM) outcomes demonstrated a significant 39% risk reduction of the primary composite endpoint (sustained ≥50% eGFR decline, ESKD, or renal or cardiovascular deaths) with dapagliflozin (10 mg/day) as compared to placebo (p<0.001). There was a 36% and 50% risk reduction of the primary composite endpoint in T2DM and non-diabetic patients, respectively. Similarly, dapagliflozin achieved a 44% risk reduction of the secondary composite endpoint of ≥50% decline in eGFR, ESKD, or renal deaths (p<0.001) and a 31% risk reduction of all-cause mortality (p=0.004) than with placebo. This study’s outcomes exhibit a marked benefit of dapagliflozin by slowing down CKD progression and reducing adverse renal events in CKD patients with or without T2DM. Consistent renoprotective effects of dapagliflozin were also observed in varying baseline albumin-to-creatinine ratio albuminuria (UACR 200-5000 mg/kg) and eGFR (<45 or >45 mL/min/1.73 m^2^) [8,10,13].

Another prespecified analysis of the DAPA-CKD study was conducted in 624 patients with stage 4 CKD (UACR 200-5000 mg/g). Dapagliflozin showed a 27% risk reduction in the primary composite endpoint and 29%, 17%, and 32% risk reductions in kidney, cardiovascular, and mortality endpoints, respectively, relative to placebo. The eGFR slope declined by 2.15 and 3.38 ml/min/1.73 m^2^ per year in the dapagliflozin and placebo groups, respectively (p=0.005). This prespecified analysis concluded no increased risks and consistent effects of dapagliflozin in stage 4 CKD and albuminuria patients with or without T2DM [14].

The most common type of glomerulonephritis is IgA nephropathy, which often progresses to kidney failure despite receiving RAAS blockers and immunosuppressants. An interesting sub-analysis of the DAPA-CKD study assessed the effectiveness of dapagliflozin in 270 biopsy-proven IgA nephropathy patients with mean eGFR of 43.8 mL/min/1.73 m^2^ and UACR of 900 mg/g. Dapagliflozin reduced the risk of the primary composite outcome by 71% and a secondary composite of the renal outcome by 75%. During the follow-up, dapagliflozin experienced a UACR reduction of 26% and a slower CKD progression, with an eGFR difference of 2.4 mL/min/1.73 m^2^/year relative to placebo. The mean rate of eGFR decline was -3.5 mL/min/1.73 m^2^/year with dapagliflozin as compared to -4.7 mL/min/1.73 m^2^/year with placebo. This subanalysis study demonstrated that dapagliflozin significantly reduced CKD risk progression and was also well tolerated in IgA nephropathy [15].

Another prespecified analysis of the DAPA-CKD study assessed the effectiveness of dapagliflozin 10 mg in 104 patients with only biopsy-confirmed FSGS (eGFR 25-75 mL/min/1.73 m^2^ and UACR 200-5000 mg/g). This study outcomes showed marked acute eGFR reduction with dapagliflozin than with placebo (−4.5, 95% confidence interval (CI) −5.9 to −3.1 vs. −0.9, −2.1 to 0.4 mL/min/1.73 m^2^/2 weeks) Subsequently, there was a decrease in mean chronic eGFR decline rates with dapagliflozin (−1.9) versus placebo (−4 mL/min/1.73 m^2^/year). Moreover, dapagliflozin was well tolerated without any case of diabetic ketoacidosis. Thus, it was concluded that dapagliflozin safely decreased the chronic eGFR decline rate over a median of 2.4 years when combined with RAAS blockers in FSGS patients [16].

The DECLARE-TIMI 58 (Dapagliflozin Effect on Cardiovascular Events - Thrombolysis in Myocardial Infarction 58) study also compared the effectiveness of dapagliflozin with placebo in DKD or NDKD. Dapagliflozin demonstrated a significantly lower risk of a sustained eGFR decline by at least 40% to eGFR 60 mL/min/1.73 m^2^ than with placebo (p<0.0001). Moreover, fewer patients in the dapagliflozin group experienced ESKD than in the placebo group (p=0.012). There was a significant improvement in long-term changes in UACR with dapagliflozin versus placebo (p<0.0001) regardless of UACR or eGFR at baseline. Dapagliflozin treatment achieved a significant decrease in composite cardiorenal outcome across subgroups of UACR ≥30 mg/g (p=0.0125) and in renal-specific outcomes across all UACR categories (p<0.05).

Positive clinical outcomes of SGLT2i in CKD patients were demonstrated with respect to the progression of renal disease, heart failure hospitalizations or cardiovascular deaths, and AKI in a recent meta-analysis study of 13 large randomized clinical trials [17].

The UK Kidney Association Clinical Practice Guideline (2023) has recommended SGLT2i as a therapeutic option in CKD patients both in and without T2DM. In patients with T2DM, SGLT2i can be initiated in CKD patients when eGFR = 20-45 mL/min/1.73 m^2^ and UACR ≥25 mg/mmol. In patients without T2DM, SGLT2i can be initiated in CKD patients when eGFR ≥20 mL/min/1.73 m^2^ and UACR ≥25 mg/mmol. The latest Kidney Disease Improving Global Outcomes (2022) has recommended SGLT2i as the preferred first-line pharmacologic therapy for patients with T2DM and CKD, regardless of their glycemic control [18].

In short, dapagliflozin is the SGLT2i drug class approved for use in both diabetic and NDKD patients. Results from randomized controlled trials on SGLT2i have determined a paradigm shift in the treatment of NDKD patients. The DAPA-CKD trial could answer the key question of whether the nephroprotection conferred by SLGT2i could extend also to CKD patients without diabetes. This case series presents data on dapagliflozin use in patient profiles of kidney diseases due to causes other than T2DM in real-world practice. The nephroprotective effect of dapagliflozin reported in clinical studies was also evident in this case series, thus highlighting dapagliflozin as an SGLT2i drug by reducing the CKD progression in patients with NDKD [8-10].

## Conclusions

This case series has demonstrated the advantages of reducing renal risk factors in NDKD patients, aligning with published data and the DAPA-CKD study. It supports the notion that dapagliflozin can effectively reduce the risk of renal disease progression in NDKD patients. Thus, it is crucial for clinicians to recognize dapagliflozin (SGLT2i) as a significant therapeutic choice in the treatment of NDKD.
